# Barriers in managing psychiatric disorders in athletes

**DOI:** 10.1192/j.eurpsy.2021.1063

**Published:** 2021-08-13

**Authors:** K. Shah, M. Ghouse, D. Kamrai

**Affiliations:** 1 Department Of Psychiatry, Griffin Memorial Hospital, Norman, United States of America; 2 Psychiatry, Griffin Memorial Hospital, Norman, United States of America

**Keywords:** sports psychiatry, athletes, diagnostic barriers

## Abstract

**Introduction:**

Athletes have participated in sports and physical exercise for several decades as a coping strategy to alleviate mental health and behavioral issues. The increasing prevalence of psychiatric disorders among athletes attributed to the failure of its appropriate management.

**Objectives:**

Our goal is to identify barriers in diagnosing and treating psychiatric problems among sportspersons to educate clinicians about the potential risk factors for athletes’ mental health disorders to provide optimal medical care.

**Methods:**

We examined MeSH terms “Athletes,” “Sports,” “Risk Factors,” “Diagnosis,” and “Patient Care Management,” in the context of “Mental Health,” “Mental Disorders,” “sports psychiatry,” and “diagnostic barriers.” We included 23 studies per the PRISMA guidelines, searching Medline, PubMed, PubMed Central, and PsychInfo databases until August 2020.

**Results:**

Barriers managing psychiatric disorders in athletes are overtraining syndrome, compensatory training, idolizing, negative coping mechanisms, social stigma, injuries, and performance-enhancing supplements usage. Other factors attributed to diagnostic barriers are general perceptions, age, racial and gender disparities, poor health services, interpersonal issues, patient-therapist relationships, sense of entitlement, control or confidentiality problems, and lack of quality preventative measures. Risk factors are injuries, sports type, doping, substance abuse, lifestyle, failures in achievement, eating disorders, and maladaptive coping mechanisms.

**Conclusions:**

These barriers in psychiatric care have adversely impacted the mental health of sportspersons. Athletes have deviated from their careers and lost valuable periods of their lives due to inadequate attention to sports psychiatry aspects, such as cognitive health services, inclusive sports management measures, diagnostic and treatment approaches, reliable mental health services, and public awareness programs.

